# The effect of a novel glycolysis-related gene signature on progression, prognosis and immune microenvironment of renal cell carcinoma

**DOI:** 10.1186/s12885-020-07702-7

**Published:** 2020-12-07

**Authors:** Fangshi Xu, Yibing Guan, Li Xue, Shanlong Huang, Ke Gao, Zhen Yang, Tie Chong

**Affiliations:** 1 Department of Medicine, Xi’an Jiaotong University, No. 76, Yanta West Road, Xi’an, 710061 Shaanxi China; 2 Department of Urology, The Second Affiliated Hospital of Xi’an Jiaotong University, No. 157, West Five Road, Xi’an, 710000 Shaanxi China; 3 Department of Emergency, The Second Affiliated Hospital of Xi’an Jiaotong University, No. 157, West Five Road, Xi’an, 710000 Shaanxi China

**Keywords:** Renal cell carcinoma, Glycolysis, Risk signature, Prognosis, Immune microenvironment, GSEA, TCGA, TIMER

## Abstract

**Background:**

Glycolysis is a central metabolic pathway for tumor cells. However, the potential roles of glycolysis-related genes in renal cell carcinoma (RCC) have not been investigated.

**Methods:**

Seven glycolysis-related gene sets were selected from MSigDB and were analyzed through GSEA. Using TCGA database, the glycolysis-related gene signature was constructed. Prognostic analyses were based on the Kaplan–Meier method. The cBioPortal database was employed to perform the mutation analyses. The CIBERSORT algorithm and TIMER database were used to determine the immunological effect of glycolytic gene signature. The expressions in protein level of eight glycolytic risk genes were determined by HPA database. Finally, qPCR, MTT and Transwell invasion assays were conducted to validate the roles of core glycolytic risk genes (CD44, PLOD1 and PLOD2) in RCC.

**Results:**

Four glycolysis-related gene sets were significantly enriched in RCC samples. The glycolytic risk signature was constructed (including CD44, PLOD2, KIF20A, IDUA, PLOD1, HMMR, DEPDC1 and ANKZF1) and identified as an independent RCC prognostic factor (HR = 1.204). Moreover, genetic alterations of glycolytic risk genes were uncommon in RCC (10.5%) and glycolytic risk signature can partially affect immune microenvironment of RCC. Six glycolytic risk genes (except for IDUA and HMMR) were over-expression in A498 and 786-O renal cancer cells through qPCR test. MTT and Transwell assays revealed that silencing of CD44, PLOD1 and PLOD2 suppressed the proliferation and invasion of renal cancer cells.

**Conclusions:**

The glycolysis-related risk signature is closely associated with RCC prognosis, progression and immune microenvironment. CD44, PLOD1 and PLOD2 may serve as RCC oncogenes.

**Supplementary Information:**

The online version contains supplementary material available at 10.1186/s12885-020-07702-7.

## Background

Renal cell carcinoma (RCC) is the third most common genitourinary cancer, accounting for approximatively 2.2% of new cancer cases worldwide [1]. It has a high mortality rate, which results in almost 200,000 deaths/year, accounting for 1.8% of all cancer-related deaths [2]. Radical nephrectomy is the main treatment for early stage RCC patients, with a 72% 5-year overall survival rate (OSR) [3]. Unfortunately, approximatively 20–30% of patients have metastatic symptoms at the time of diagnosis, with only a 12% 5-year OSR [4]. In addition, 20–30% of patients diagnosed with T1–2 stages suffered from metastasis within 1 to 2 years after surgery [5]. Although molecular-targeted drugs improve survival time of metastatic patients, the median survival time is still less than 3 years [6]. Therefore, it is still urgent and challenging to explore RCC molecular mechanisms and find potential therapeutic targets and prognostic biomarkers.

With the last decade progress in oncology, the reprogramming of energy metabolism has been considered as a new hallmark of cancers [7]. Particularly, the tumor cells’ carbohydrate metabolism pattern was found to be extremely different from that of normal cells. Even in the presence of abundant oxygen, cancer cells still largely switch their metabolism to glycolysis, a metabolic process called Warburg effect [7]. Glycolysis is an enzymatic reaction, that is collectively regulated by 3 limiting enzymes (including HK,PFK and PKM2) and some equilibrium enzymes of fructose-bisphosphatase (including ALDO, ENO, GAPDH) [8]. Glycolysis-related genes, which encode these regulatory enzymes, were shown to be closely related to malignant progression and prognosis of many cancers, and therefore, are considered as potential therapeutic targets [9]. Hexokinase (HK) is overexpressed in rectum [10], breast [11] and gastric cancers [12] and is associated with poor prognosis. Phosphofructokinase 1(PFK1), a key enzyme in glycolysis flux control [13], is also up-regulated in breast cancer and can promote malignant proliferation and invasion through p-AKT [14] activation. However, the potential effect of glycolytic genes on RCC prognosis and progression has not been investigated. Besides, the role of glycolysis in RCC is also not fully elucidated.

With the continuous development of bioinformatics and high-throughput technology, several biomarkers have been found to be closely associated with RCC prognosis and malignant progression [15]. However, the predictive ability of single-gene biomarkers is limited in RCC prognosis analysis and multiple-genes signature maybe a better strategy [16]. So far, only four studies have established the glycolytic risk signature in lung adenocarcinoma [17], endometrial cancer [16], hepatocellular carcinoma [18] and bladder cancer [19]. Therefore, to explore the potential roles of glycolytic metabolism and glycolysis-related genes in RCC, a series of bioinformatic analyses were performed through TCGA database (609 samples). We found that glycolysis is significantly enriched in RCC and constructed a glycolysis-related risk signature. The novel risk signature was closely associated with RCC prognosis, progression and immune microenvironment. Moreover, its hub genes (CD44, PLOD1 and PLOD2) were proven to possess pro-cancer abilities through MTT and Transwell invasion assays. Our research provides a new method for RCC prognostic analysis and a new insight into the mechanisms of glycolytic genes in RCC progression.

## Methods

### Date source

The gene expression data and corresponding clinical information were obtained from the Cancer Genome Atlas (TCGA) public database. A total of 609 samples were analyzed, including 537 RCC and 72 normal or paracancerous samples. The clinical features of RCC patients were shown in Table 1. The data type of gene expression was transcriptome profiling and Gene Expression Quantification, and the type of clinical data was BCR-XML. The selected sample types were adenomas and adenocarcinomas.

**Table 1:** Clinical characteristics of 537 RCC patients included in present study from TCGA database

Variables	Number (percentage)
Vital status	
Alive	367 (68.3%)
Dead	170 (31.7%)
Age	
<65	336 (62.6%)
≥ 65	201 (37.4%)
Gender	
Male	346 (64.4%)
Female	191 (35.6%)
Tumor Grade	
G1	14 (2.6%)
G2	230 (42.8%)
G3	207 (38.5%)
G4	78 (14.5%)
Gx	5 (1.0%)
Unknow	3 (0.6%)
Clinical Stage	
Stage I	269 (50.1%)
Stage II	57 (10.6%)
Stage III	125 (23.3%)
Stage IV	83 (15.4%)
Unknow	3 (0.6%)
T stage	
T1	275 (51.2%)
T2	69 (12.8%)
T3	182 (33.9%)
T4	11 (2.1%)
M stage	
M0	426 (79.3%)
M1	79 (14.7%)
Mx	30 (5.6%)
Unknow	2 (0.4%)
N stage	
N0	240 (44.7%)
N1	17 (3.2%)
Nx	280 (52.1%)
Survival time (day) (170 dead samples)	
≤ 365	52 (30.6%)
365<ST ≤ 730	33 (19.4%)
730<ST ≤ 1095	28 (16.5%)
≥ 1095	57 (33.5%)
Follow up (day) (367 alive samples)	
≤ 365	66 (18.0%)
365<FT ≤ 730	59 (16.1%)
730<FT ≤ 1095	33 (9%)
≥ 1095	209 (56.9%)

*ST* survival time, *FT* follow up time

### Glycolysis-related gene sets and GSEA

Well-annotated gene sets, representing the universe of the biological processes, are critical for meaningful and insightful interpretation of large-scale genomic data [20]. The Molecular Signatures Database (MSigDB) is a collection of annotated gene sets for GSEA software use (https://www.gsea-msigdb.org/gsea/msigdb/index.jsp) [21]. Combination with the selecting strategies of previous glycolysis-related studies [22–24], we used ‘glycolysis’ and ‘glycolytic’ as the searching terms in the MSigDB database and furtherly screened out seven priori glycolysis-related gene sets. These gene sets included Hallmark Glycolysis, Go Glycolytic Process, KEGG Glycolysis Gluconeogenesis, Reactome Glycolysis, BioCarta Feeder Pathway and Module 306. The descriptions of these gene sets were shown in Supplementary Table 1.

GSEA is a computational method that determines whether an a priori defined set of genes shows statistically significant and concordant differences between two biological phenotypes [25]. Enrichment statistics was based on weighted method. Phenotype labels were set as tumor versus normal samples. Permutation Type was phenotype. The number of permutations was set as 1000 and the gene sets were considered to significantly enriched in RCC samples, when the normalized enrichment score (NES) ≥1, nominal (NOM) *p*-value ≤0.05 and false discovery rate (FDR) *q*-value ≤0.25 were simultaneously satisfied. The detailed parameter settings of GSEA were presented in Supplementary Table 2.

### Identification and functional analyses of glycolysis-related DEGs

TCGA data extraction and arrangement were performed by Perl (Practical Extraction and Report Language) version 5.28. The differential expression of glycolysis-related genes between tumor and normal samples was analyzed via the ‘Limma’ package in R software Ver3.6.2, in order to select differentially expressed genes (DEGs). When *p*-value < 0.05 and Log_2_FC absolute value ≥1, the gene was regarded as differentially expressive in tumor samples. Protein-protein interaction (PPI) networks of glycolysis-related genes were constructed using the String database [26] and Cytoscape software Ver3.4.0 [27].

To explore the biological function of glycolysis-related DEGs in RCC, we performed Gene Ontology (Go) and Kyoto Encyclopedia of Genes and Genomes (KEGG) enrichment analyses through David database [28]. The figures were drawn by the ggplot2 package (R software). The bioinformatics analysis of Go consisted in three categories, namely molecular function (MF), biological process (BP) and cellular components (CC). The pathways and biological functions with top ten enriched gene counts were selected for graphing.

### Construction of the risk signature

Cox univariate analysis was used to preliminarily screen out the glycolysis-related genes which could affect RCC prognosis. Subsequently, the risk signature based on glycolysis-related genes was established through multivariate regression analysis. Since the expression levels of glycolysis-related genes, in the risk signature, were all high, Log2 transformation was used to normalize the expression profiles of these genes, which can prevent the generation of diminutive coefficients. The risk plot and the corresponding heatmap were drawn using the ‘pheatmap’ package in R software.

### Prognosis analyses

According to the glycolytic risk signature, the risk score of each RCC samples was further calculated. Subsequently, RCC samples were divided into high- and low-risk groups, according to the median of risk score. The prognostic difference between high- and low-risk groups was evaluated via the ‘survival’ package in R software. Moreover, cox univariate and multivariate analyses were successively performed to identify whether risk score was an RCC independent prognostic factor. To assess the applicative scope of glycolytic risk signature in RCC prognosis analysis, the prognostic difference was detected among RCC patients between different risk groups under the same clinical subgroup.

Receiver operating characteristic curve (ROC) was employed to assess predicting accuracy of glycolytic risk signature in RCC prognosis analysis. Decision curve analysis (DCA) was used to evaluate the clinical net benefit (NB) brought by the novel signature [29].

### Mutation analyses of glycolysis-related genes

To better comprehend the genomics profiles of glycolysis-related genes, we performed mutation analyses using the cBioPortal online database [30]. The OncoPrint tab summarizes genomic alterations in all queried genes across a sample set [31]. The cancer types summary tab reveals mutation types and frequency information of queried genes.

### Immune analyses of glycolysis-related genes

To determine the effect of glycolysis-related risk signature on immune microenvironment of RCC, immune infiltration analyses were performed through the CIBERSORT algorithm and TIMER database. The immune abundances of 22 leukocyte subtypes in each RCC samples were obtained by using the CIBERSORT algorithm. The landscape of immune distributions of each RCC samples were presented by ‘barplot’ package. The differential infiltration levels of 22 immune cells between high- and low-risk groups were visualized via the ‘vioplot’ package in R software.

The public database, Tumor Immune Estimation Resource (TIMER) (https://cistrome.shinyapps.io/timer/), was employed to assess the relationships between the somatic copy number alteration (SCNA) of eight risk signature genes and the infiltration levels of six immune cells (including B cells, CD4 + T cells, CD8 + T cells, neurphils, macrophases and dendritic cells).

### Protein levels of glycolytic risk genes in the HPA database

The Human Protein Atlas (HPA) is a database with the aim to map all the human proteins in cells, tissues and organs using integration of various omics technologies (https://www.proteinatlas.org/) [32]. The HPA database consists of six separate parts. Among that, the Tissue and the Pathology Atlas provide information regarding the expression profiles of specified genes in normal and tumor tissues on protein levels. All images of tissues in HPA database are stained by immunohistochemistry.

### Cell culture

Two renal cancer cell lines (786-O and A498) and one normal human renal tubular epithelial cell line (HK-2) were purchased from the Institute of Biochemistry and Cell Biology at the Chinese Academy of Sciences (Shanghai, China). Cancer cells were cultured in RPMI-1640 medium (GIBCO-BRL, Invitrogen, Carlsbad, CA, USA) with 10% fetal bovine serum (FBS, Hyclone, Logan, Utah, USA). HK-2 cells were cultured in keratinocyte medium (KM, ScienCell, San Diego, California, USA) plus 1% keratinocyte growth supplement (KGS, Scien-Cell, San Diego, California, USA). All mediums were treated with 100 U/ml penicillin and 100μg/ml streptomycinm, and all cells were incubated at 37 °C with 5% CO 2.

### Quantitative real-time PCR (qRT-PCR)

Total RNA was extracted using TRIzol reagent (G-Clone, Beijing, China). The cDNA was synthesized with the PrimeScript RT reagent Kit with gDNA Eraser (Takara, Japan). qRT-PCR was performed using the TB Green Premix Ex Taq II (Takara, Japan) in ABI 7500 Real-Time PCR instrument. The primers were listed in Supplementary Table 3. GAPDH was used as an internal control. The relative mRNA levels were calculated based on 2^−ΔΔCt^ method.

### Cell transfection

The expression in mRNA level of CD44, PLOD1 and PLOD2 were silenced by specific small interfering RNAs (siRNAs). Si-CD44, si-PLOD1, si-PLOD2 and si-NC (negative control) were synthesized by GenePharma Biotechnology (Shanghai, China). 786-O and A498 cells were transfected by these siRNAs through the Lipofectamine 2000 (Invitrogen, Thermo Fisher, Waltham, MA, USA) Further experiments were performed 48 h after transfection.

### MTT assay

Transfected cells were seeded into 96-well plate (5 × 10^3^ cells per well) and incubated for 24, 48 and 72 h. Every detecting point (24 h/each time), 10 μL MTT reagent (Life science, NY, USA) was added to each well and incubated for 4 h. After removing the medium and washing each well by PBS, 100 μL DMSO was added into each well and the absorbance at 490 nm was measured by a microplate reader (ThermoFisher, Waltham, MA, USA).

### Transwell invasion assay

The polycarbonate membrane was coated with the matrigel (200 ng/mL; BD Biosciences, Franklin Lakes, NJ, USA). Then transfected cancer cells were seeded with serum-free RPMI-1640 medium into the upper chambers (1 × 10^4^ cells/well). The RPMI-1640 medium with 10% FBS was added into the bottom chambers. After 24 h incubation at 37 °C, the invasive cells adhering to the lower surface of the membrane were fixed by 4% paraformaldehyde and stained with 0.1% crystal violet. The invasive cells were photographed by a microscope.

### Statistical analysis

All statistical analyses and graphing were performed by the R software (version 3.6.2) or GraphPad Prism (version 8.0.1). The Student’s t test was used to compare the expressive difference of glycolysis-related genes. The Kolmogorov–Smirnov test was used to evaluate the relationships between glycolytic risk score and RCC clinicopathological characteristics. Additionally, the survival analysis was based on the Kaplan–Meier method. A *p*-value < 0.05 was considered significant.

## Results

### Glycolytic metabolism is significantly enriched in RCC samples

**Fig. 1:**
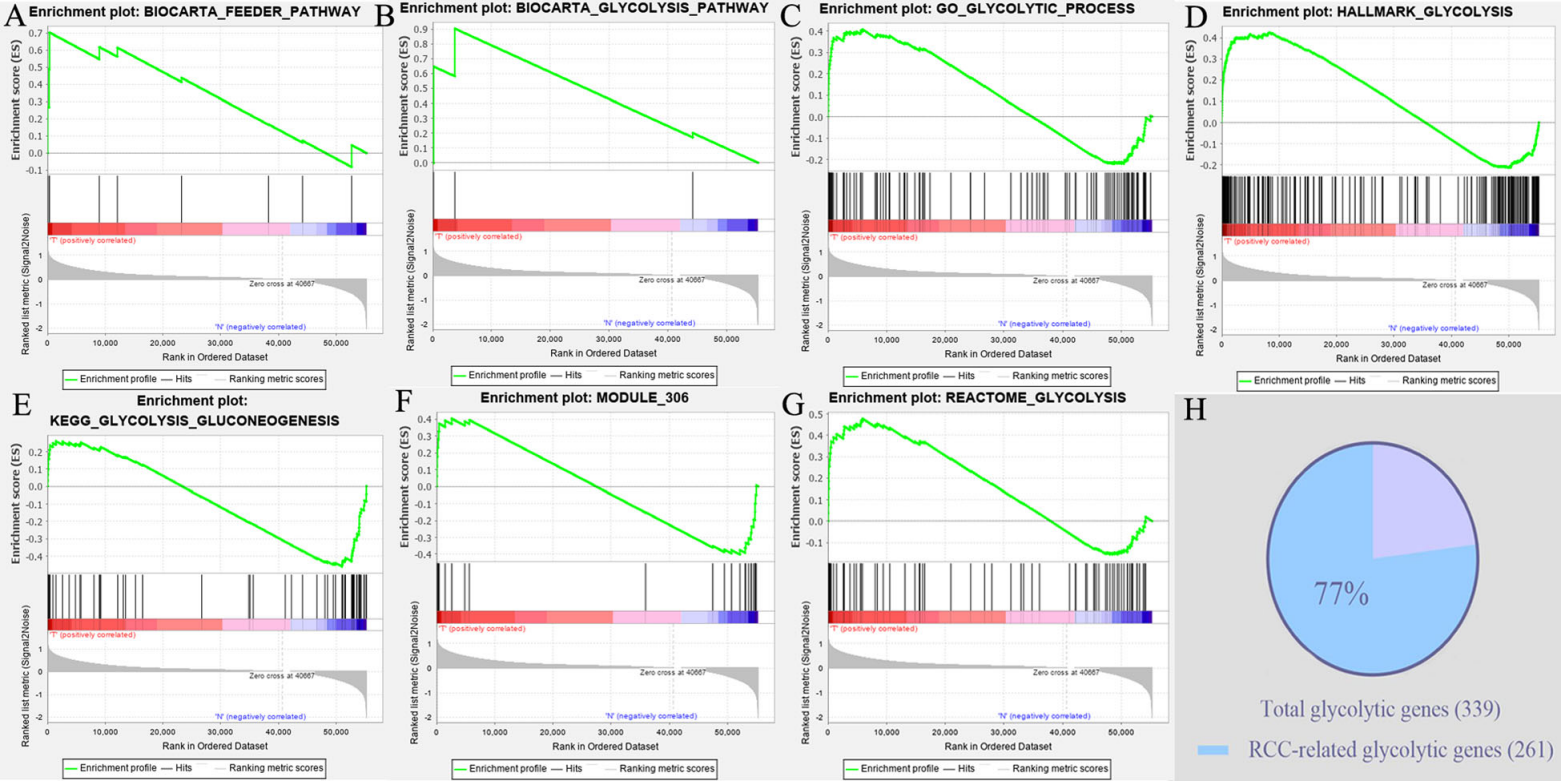
Enrichment plots of seven gene sets by performing GSEA. **a**-**g** The analytical results of GSEA. **h** Pie Graph of enriched glycolysis-related genes. GSEA, Gene-set enrichment analysis

Five hundred thirty-seven RCC samples from TCGA database were included in our bioinformatic analyses (Table 1). Seven glycolysis-related gene sets were selected from the MSigDB for GSEA and the analytical results were shown in Fig. 1. Four gene sets were significantly enriched in RCC samples, namely Biocarta feeder pathway, Biocarta glycolysis pathway, Reactome glycolysis and Hallmark glycolysis (Table 2). Glycolysis-related gene sets (44.4%, 4/9) and glycolysis-related genes (77.0%, 261/339) were greatly enriched in RCC, suggesting that the glycolytic metabolism was prevalent in RCC, which is consistent with the general metabolic hallmark of cancers. After the GSEA preliminary screening, a total of 261 glycolysis-related genes from the 4 enriched gene sets were speculated to play important roles in RCC and were therefore selected for further analyses.

**Table 2:** The GSEA results of glycolysis-related Gene sets in RCC (611 samples)

Name of GS	Size	NES	NOM p-val	FDR q-val
Biocarta feeder pathway	9	1.59	0.037	0.037
Biocarta glycolysis pathway	3	1.62	0.031	0.031
Reactome glycolysis	72	1.44	0.047	0.047
GO glycolytic process	106	1.46	0.084	0.084
Hallmark glycolysis	200	1.67	0.038	0.038
KEGG glycolysis gluconeogenesis	62	−1.42	0.146	0.146
Module 306	26	0.99	0.458	0.458

*GS* gene set, *NES* normalized enrichment score, *NOM p-val* nominal p-value, FDR q-val false discovery rate q-value

### Glycolysis-related genes are differential expressed in RCC samples and regulate some cancer-related biofunction

**Fig. 2:**
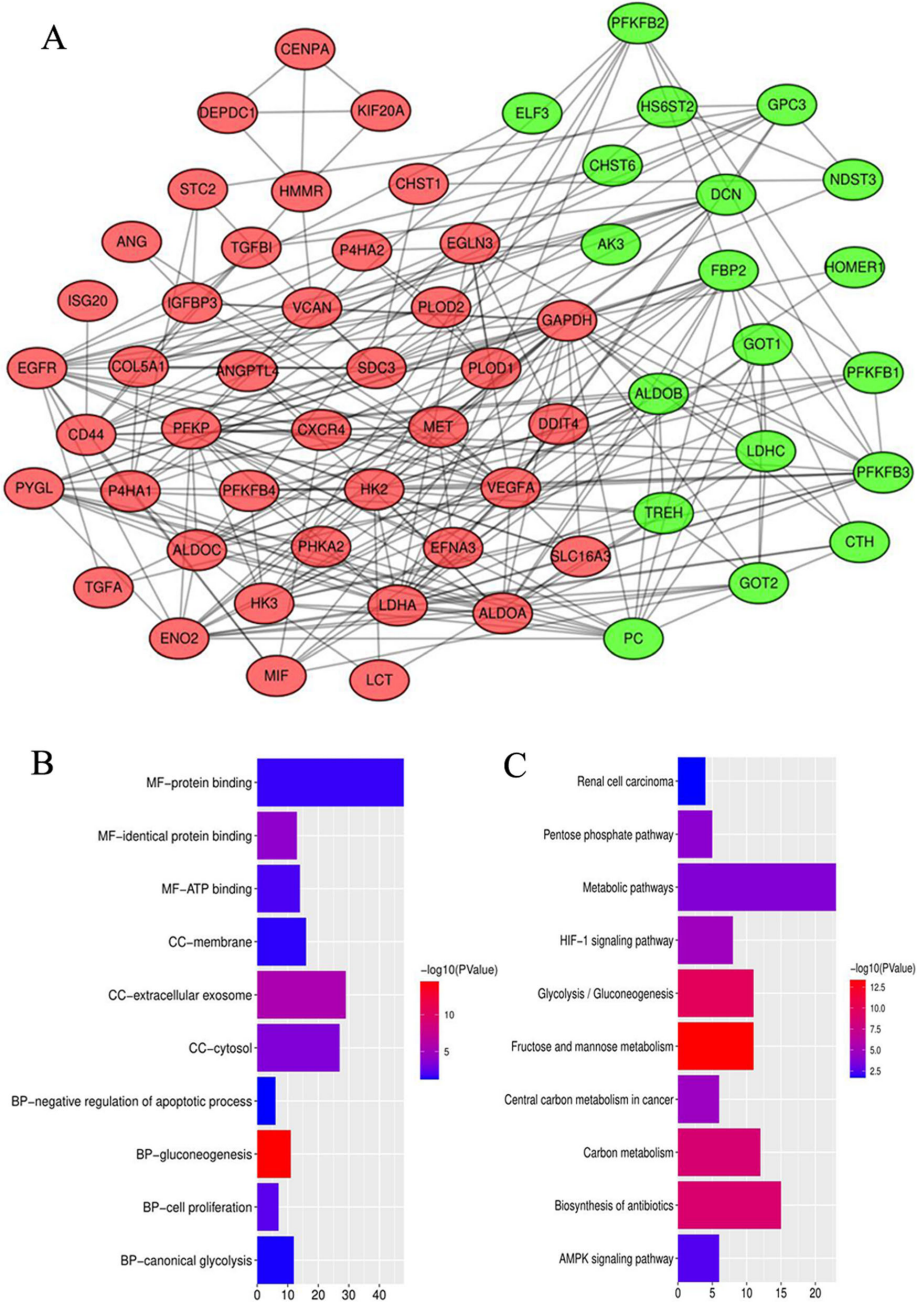
Identification and functional analyses of glycolytic-related DEGs. **a** The PPI network of 75 glycolysis-related DEGs. When absolute value of log2FC ≥ 1 and adjusted *p* < 0.05 were simultaneously obtained, the expression of glycolysis-related genes was considered as significantly differential in tumor samples. Up-regulated and down-regulated genes are red and green, respectively. **b** Go enrichment analysis of 75 glycolysis-related DEGs. **c** KEGG enrichment analysis of 75 glycolysis-related DEGs. PPI, protein-protein interaction; DEGs, differential expression genes; MF, molecular function; BP, biological process; CC, cellular components

To further screen out the hub genes that contribute to RCC malignant progression, we assessed the differential expression of glycolysis-related genes between RCC and normal samples. A total of 75 out of 261 glycolytic genes were significantly differentially expressed in RCC. The PPI network of 75 glycolysis-related DEGs was constructed via the String database and Cytoscape software (Fig. 2a). Among three kinds of glycolysis limiting enzymes, HK3 was up-regulated in RCC, while the expression of PKM family members were bidirectional regulated (PFKP and PFKFB4 were up-regulated; PFKFB1–3 were down-regulated). However, there was no expressive difference of PKM between RCC and normal samples.

GO enrichment analysis revealed that the subcellular functional localization of glycolytic genes was cytoplasm, and these genes may regulate glycolytic metabolism through the exosome pathway (Fig. 2b). Meanwhile, these glycolytic genes also engaged in cell proliferation and apoptosis. KEGG enrichment analysis showed that glycolysis-related genes not only involved in glycolysis, pentose phosphate and fructose metabolism pathways, but also were closely related to some cancer-related signal pathways, such as HIF-1, renal cell carcinoma and AMPK pathways (Fig. 2c).

### Eight glycolytic DEGs constitute the glycolysis-related risk signature in RCC

**Fig. 3:**
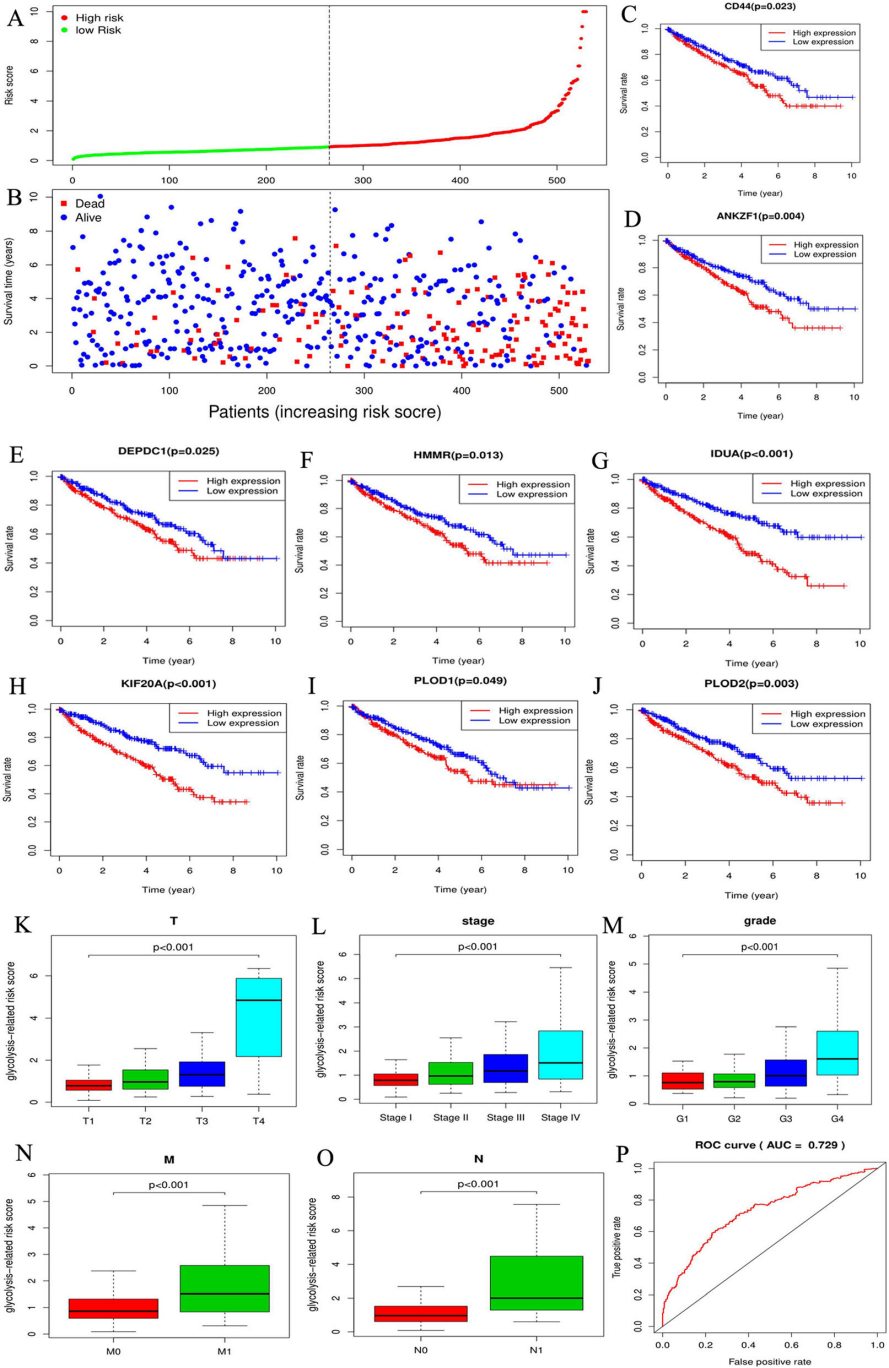
Construction of glycolysis-related gene signature. **a** Risk plot. The curve shows the risk score distribution of 539 RCC samples. The X-axis represents the ranking of 539 RCC patients from low to high according to their own risk score. The y-axis represents the risk score of each RCC patient. The dotted line represents median of risk score. (**b**) Survival status. The dotted line and X-axis in this graph have the same meaning as the risk plot. The y-axis represents the survival time of RCC patients. The different colors of dot represent different survival status. **c**-**j** Survival analyses of eight signature genes. **k**-**o** The relationships between risk score and RCC clinicopathological features. RCC, renal cell carcinoma. **p** The ROC of glycolytic risk signature

A single gene generally cannot accurately predict cancer prognosis; however, a gene signature, consisting of multiple genes, could improve the accuracy of prognosis analysis. Through univariate regression analysis, 23 out of the 75 glycolysis-related DEGs were found to greatly affect RCC prognosis (Table 3). Subsequently, the glycolytic risk signature was constructed via multivariate regression analysis (Table 3). Risk score = 0.239 × CD44 + 0.203 × PLOD2 + 0.695 × KIF20A + 0.527 × IDU4–0.265 × PLOD1–0.998 × HMMR + 0.886 × DEPDC1 + 0.199 × ANKZF1 (the gene expression was normalized with log2 transformation). Eight risk signature genes were all over-expressed in RCC samples (Supplementary Figure 1) and their overexpression all led worse survival outcomes (Fig. 3c-j), which indicated that they may serve as oncogenes.

**Table 3:** Univariable and multivariable analyses of glycolysis-related DEGs

Gene	Univariate analysis				Multivariable analysis	
	HR	UI of 95%CI	LI of 95%CI	*p*-value	Coef	HR
CD44	1.462	1.254	1.705	1.24E-06	0.239	1.270
PLOD2	1.357	1.165	1.582	9.16E-05	0.203	1.225
KIF20A	1.946	1.634	2.316	7.19E-14	0.695	2.003
IDUA	2.061	1.643	2.586	4.2E-10	0.527	1.695
PLOD1	1.306	1.044	1.633	0.019442	−0.265	0.767
HMMR	1.878	1.486	2.373	1.27E-07	−0.998	0.369
DEPDC1	2.284	1.740	2.997	2.7E-09	0.886	2.426
ANKZF1	1.452	1.189	1.772	0.000248	0.199	1.220
RBCK1	1.925	1.571	2.358	2.65E-10	–	–
CAPN5	0.710	0.548	0.918	0.009126	–	–
COL5A1	1.332	1.179	1.505	4.25E-06	–	–
ISG20	1.695	1.328	2.162	2.17E-05	–	–
GAPDH	1.462	1.129	1.892	0.003945	–	–
TGFBI	1.127	1.052	1.206	0.000614	–	–
AK3	0.578	0.428	0.779	0.000325	–	–
ENO2	1.276	1.084	1.502	0.003391	–	–
GOT1	0.636	0.525	0.769	3.11E-06	–	–
CENPA	2.449	1.956	3.065	5.47E-15	–	–
EFNA3	1.391	1.104	1.752	0.005062	–	–
HK3	1.763	1.417	2.193	3.63E-07	–	–
ALDOB	0.859	0.801	0.921	2.2E-05	–	–
MIOX	0.866	0.807	0.929	5.86E-05	–	–
TREH	0.687	0.539	0.875	0.00231	–	–

*DEGs* differentially expressed genes, *HR* hazard ratio, *CI* confidence interval, *UI* upper limit, *LI* lower limit, *Coef* coefficient

The risk score of each RCC patient was calculated using the risk formula and the patients were divided into high-and low-risk groups according to median of risk score (Fig. 3a). The proportion of death events in high-risk group was significantly higher than that in low-risk group (Fig. 3b). Moreover, patients in high-risk group were often associated with undesirable clinicopathological features (Fig. 3k-o). These results indicate that the risk signature based on glycolytic genes not only affect RCC prognosis but also is closely associated with RCC malignant progression. Besides, glycolytic risk signature also possessed a good predictive accuracy in prognosis analysis (AUC = 0.729) (Fig. 3p).

### Glycolysis-related risk signature is valuable for RCC prognostic analyses

**Fig. 4:**
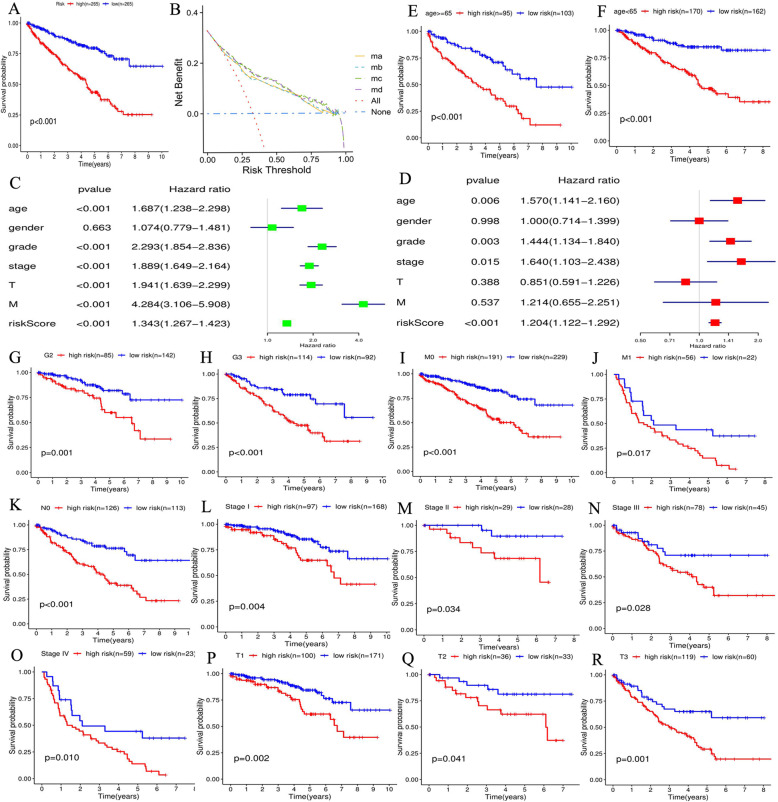
Glycolysis-related risk signature has important clinical applicable value. **a** Survival analysis between high- and low-risk groups. **b** The DCA curve of glycolytic risk signature. ma-d means four kinds of RCC prognostic models (based on multivariate Logistic regression analysis) composed of different indexes. Model a represents the prognostic model composed of age, grade and clinical stage. Model b represents the prognostic model composed age, grade and TNM stages. Model c represents the model a that added glycolytic risk score. Model d represents the model b that added glycolytic risk score. **c** The result of cox univariate analysis. **d** The result of cox multivariate analysis. **e**-**r** The verification for predictive ability of glycolytic risk signature in RCC prognostic analyses. RCC, renal cell carcinoma

Comparing with the low-risk group, the high-risk group led to worse 3-year (62.9% vs 86.2%) and 5-year overall survival rates (42.4% vs 78.2%) (Fig. 4a). Moreover, the DCA curve indicated that taking glycolysis risk signature into the RCC prognostic analysis can bring significant clinical benefit when making clinical decision (Fig. 4b).

Through univariate and multivariate regression analyses, glycolytic risk score (HR = 1.204, *p* < 0.001) was identified as an independent RCC prognostic factor (Fig. 4cd). To further determine the applicable scope of glycolytic risk signature in RCC prognostic analysis, the prognostic difference between different risk groups under the same clinical subgroup, was compared (Fig. 4g-r). Glycolytic risk signature could sensitively distinguish the prognostic differences in patients with age ≥ 65, age < 65, Grade 2–3, M0–1 stages, N0 stage, clinical stage I-IV and T1–3 stages. Due to lack of samples, glycolytic risk signature did not detect the prognostic difference of RCC patients with Grade1/4, N1 and T4 stages (Supplementary Figure 2A-D). Therefore, there is ample evidence reiterates that glycolytic risk signature is a beneficial and universally applicable method for RCC prognostic analysis.

### Genetic alteration of glycolytic risk signature is uncommon in RCC

**Fig. 5:**
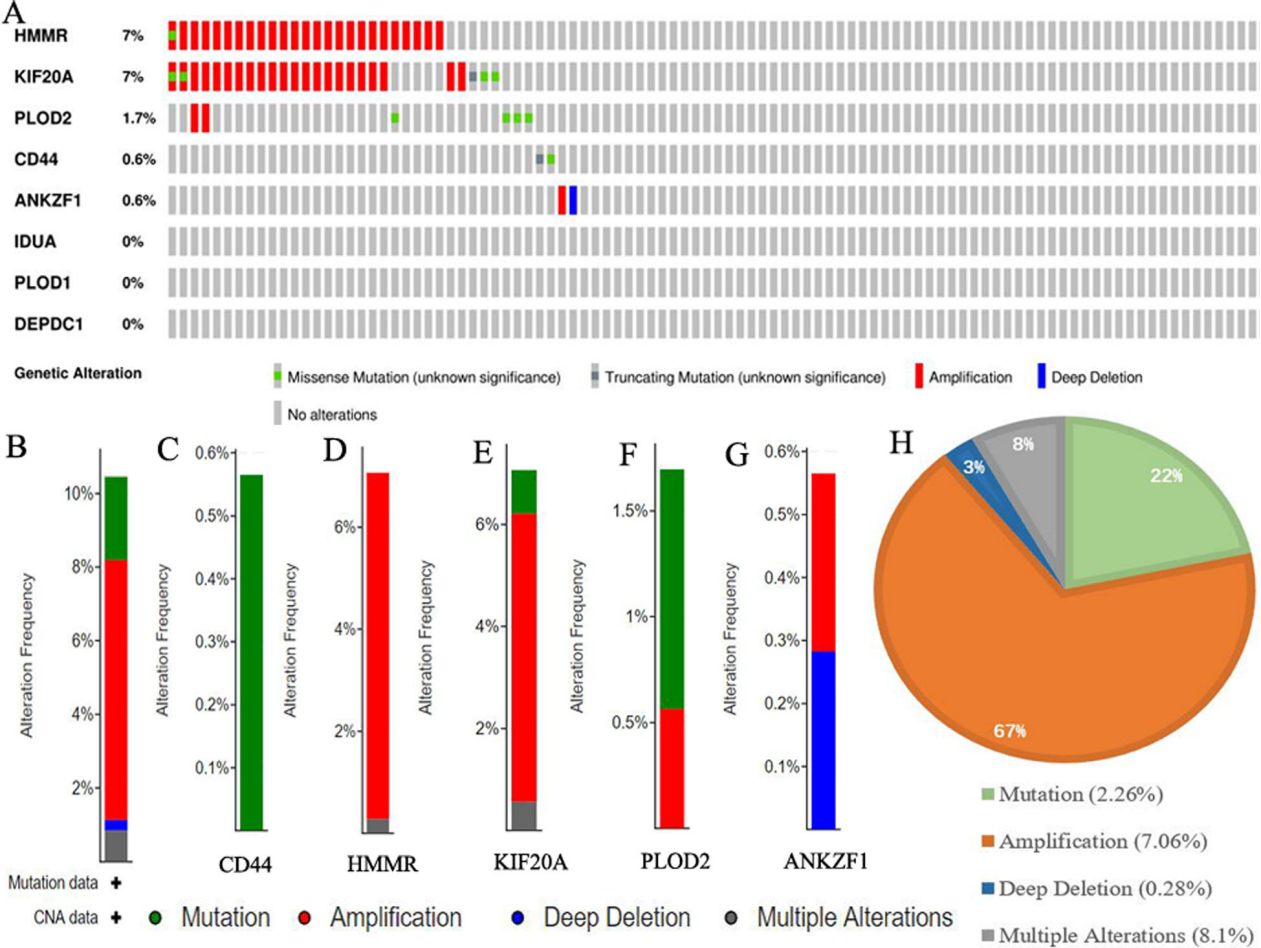
Mutation analyses of glycolysis-related risk signature. **a** The summary of genomic alterations in eight glycolysis-related genes. Each row represents a gene, and each column represents a tumor sample. Different color bars indicate different mutation types. **b**-**g** The mutation types of each genes. The y-axis represents the alteration frequency in all RCC samples. **c** Pie Graph of different mutation types. The percentage followed the mutation symbol represents the alteration frequency of the same mutational type in all RCC samples. The percentage in the pie chart represents the proportion of the same mutational type in all mutated samples. RCC, renal cell carcinoma

To better understand the genomic characteristics of glycolytic genes in RCC, the cBioPortal database was used for mutation analysis. A summary of the genetic alterations in eight risk signature genes is shown in Fig. 5a. Although these glycolysis-related genes were significantly differentially expressed in RCC samples, their mutation frequency was not high. Based on TCGA database, glycolytic genes were only mutated in 37 out of the 354 RCC samples (10.5%).

HMMR and KIF20A possessed the highest mutation frequency (7%), while IDUA, PLOD1 and DEPDCA did not generate any mutations (Fig. 5a). Moreover, we also analyzed mutational type. Different glycolytic risk genes were provided with different mutational types (Fig. 5b-g). For example, deep deletion only occurred in the genetic alterations of ANKZF1 (Fig. 5g) and CD44 harbored only one kind of alteration pattern, namely mutation (Fig. 5b). Overall, amplification was the most common mutational form (Fig. 5h) and the rarest form was deep deletion (3%). All these results revealed that the glycolytic risk signature was relatively conserved in RCC and may not reflect genetic predisposition of RCC.

### Glycolytic risk signature could affect immune microenvironment of RCC to some extent

**Fig. 6:**
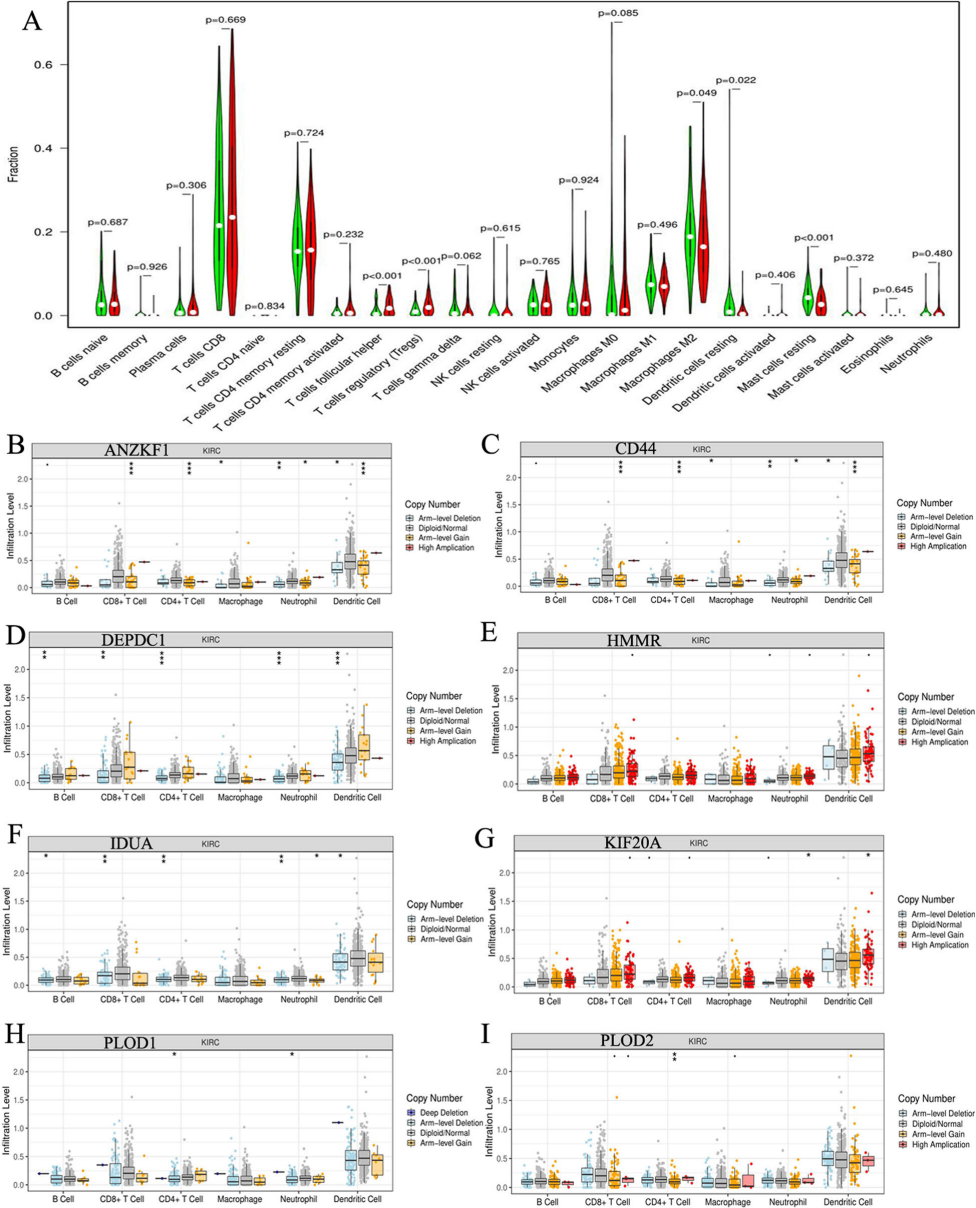
Immune infiltration analyses of glycolysis-related risk signature. **a** The differential proportion of 22 immune cells between high- and low-risk groups. High-risk group is red and low-risk group is green. **b**-**i** The relationships between SCNA of eight risk signature genes and the infiltration levels of six kinds of immune cells. RCC, renal cell carcinoma; SCNA, somatic copy number alteration; .means *p*<0.1; * means *p*<0.05; * * means *p*<0.01; * * * means *p*<0.001

We further analyzed the effect of eight glycolytic risk genes on RCC immune microenvironment. The immune abundances of 22 leukocyte subtypes in each RCC samples were exhibited in Supplementary Figure 3. The immune proportions of each sample were diverse. Moreover, the difference of immune abundance between high- and low-risk groups was determined (Fig. 6a). High risk groups brought increased infiltration levels of T cells follicular helper (*p*<0.001) and T cells regulatory (Tregs) (*p*<0.001) in RCC; Inversely, it could decrease the immune abundance of Macrophages M2 (*p* = 0.049), Dendritic cells resting (*p* = 0.022) and Mast cells resting (*p*<0.001) (Fig. 6a). However, the risk signature cannot bring any change on immune infiltration levels of CD4 and CD8 T cells. Combination with some immunological studies [33–37], the basic biofunction in immune response of these affected lymphocytes and the final effect of their alterations against antitumor immunological process were shown in Table 4. Alterations of immune abundance caused by high glycolytic risk did not always hinder antitumor immune process (Table 4).

**Table 4:** Immune effects of the lymphocytes with altered abundance

Immune cells	Alteration in high risk group	Study	Basic function	Final effect to anticancer immune
T cells follicular helper	Increased	Vinuesa CG et al. [33].	TFH can develop humoral immunity by assisting the formation of germinal center.	Promotion
T cells regulatory	Increased	Juang CM et al. [34].	Tregs are suppressive T cells and can mediate immunosuppression and tumor immune evasion.	Suppression
Macrophages M2	Decreasing	Italiani P et al. [35].	M2 cells can promote tumor cells proliferation and repair through shifting the arginine metabolism to ornithine and polyamines.	Promotion
Dendritic cells resting	Decreasing	P Brossart et al. [36].	DCs are the most potent antigen-presenting cells with the ability to stimulate naive resting T cells and to initiate primary immune responses.	Suppression
Mast cells resting	Decreasing	Dyduch G et al. [37].	Mast cells play a pro-tumor or anti-tumor role by secreting different factors (VEGF, bFGF vs TNF-α, IL-1, IL-6)	Uncertain

*TFH* T cells follicular helper, *Tregs* T cells regulatory, *DCs* Dendritic cells, *VEGF* vascular endothelial growth factor, *bFGF* basic fibroblast growth factor, *TNF-α* Tumor necrosis factor α, *IL* interleukin

Moreover, the relationships between SCNA of eight risk signature genes and the infiltration levels of six immune cells were explored through TIMER database (Fig. 6b-i). The arm-level gain in copy number of ANZKF1, CD44 and PLOD2 could lead an increment on infiltration levels of CD4 T cells (Fig. 6bci). However, as the most common mutational type (Fig. 5h), the amplification of these genes could not affect the infiltration levels of CD4 and CD8 T cells. Besides, the amplified alteration of other risk genes did not contribute to the increasing infiltration levels of macrophages, CD4 and CD8 T cells. In a word, the effect of glycolytic risk signature on immune microenvironment was limit and complex.

### Glycolytic risk genes differentially express in protein levels

**Fig. 7:**
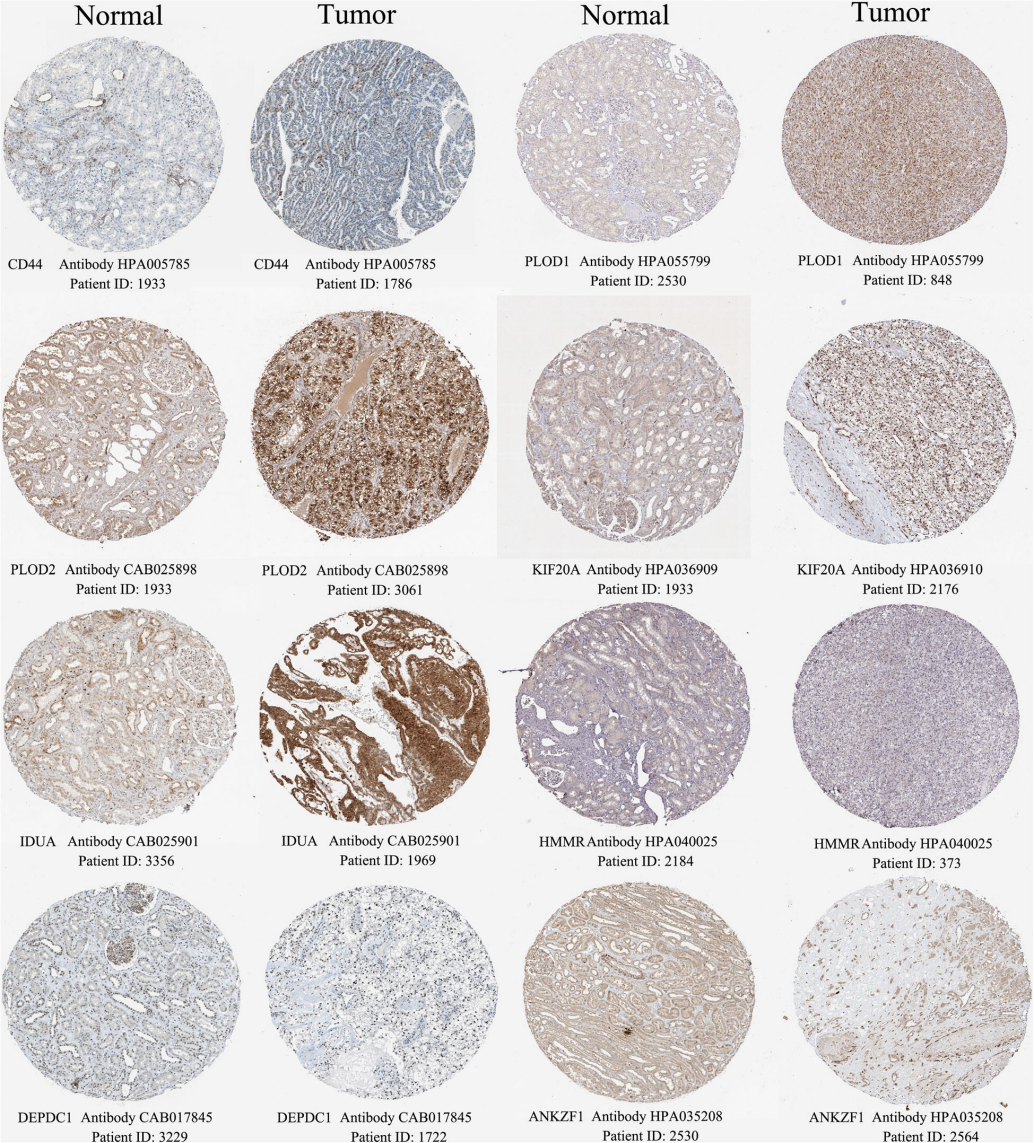
The histological expression of glycolysis-related risk genes. The top of the figure indicates the category of tissue specimen. The name of glycolytic gene, the antibody type used in immunohistochemistry, and the patient ID of tissue specimens are shown at the bottom of each image

The histological expressions of glycolysis-related risk genes in normal and tumor tissues were exhibited in Fig. 7. There was significantly expressive difference of these genes in protein levels between normal and renal cancer tissues. Among that, the protein expression of PLOD1, PLOD2, KIF20A, IDUA and DEPDC1 were obviously up-regulated; However, discernable expressive difference was not found in CD44, HMMR and ANKZF1. The protein expression of CD44 and ANKZF1 were low in both normal and tumor tissues. And that of HMMR were even not detected.

### CD44, PLOD1 and PLOD2 can promote the proliferation and invasion of renal cancer cells

**Fig. 8:**
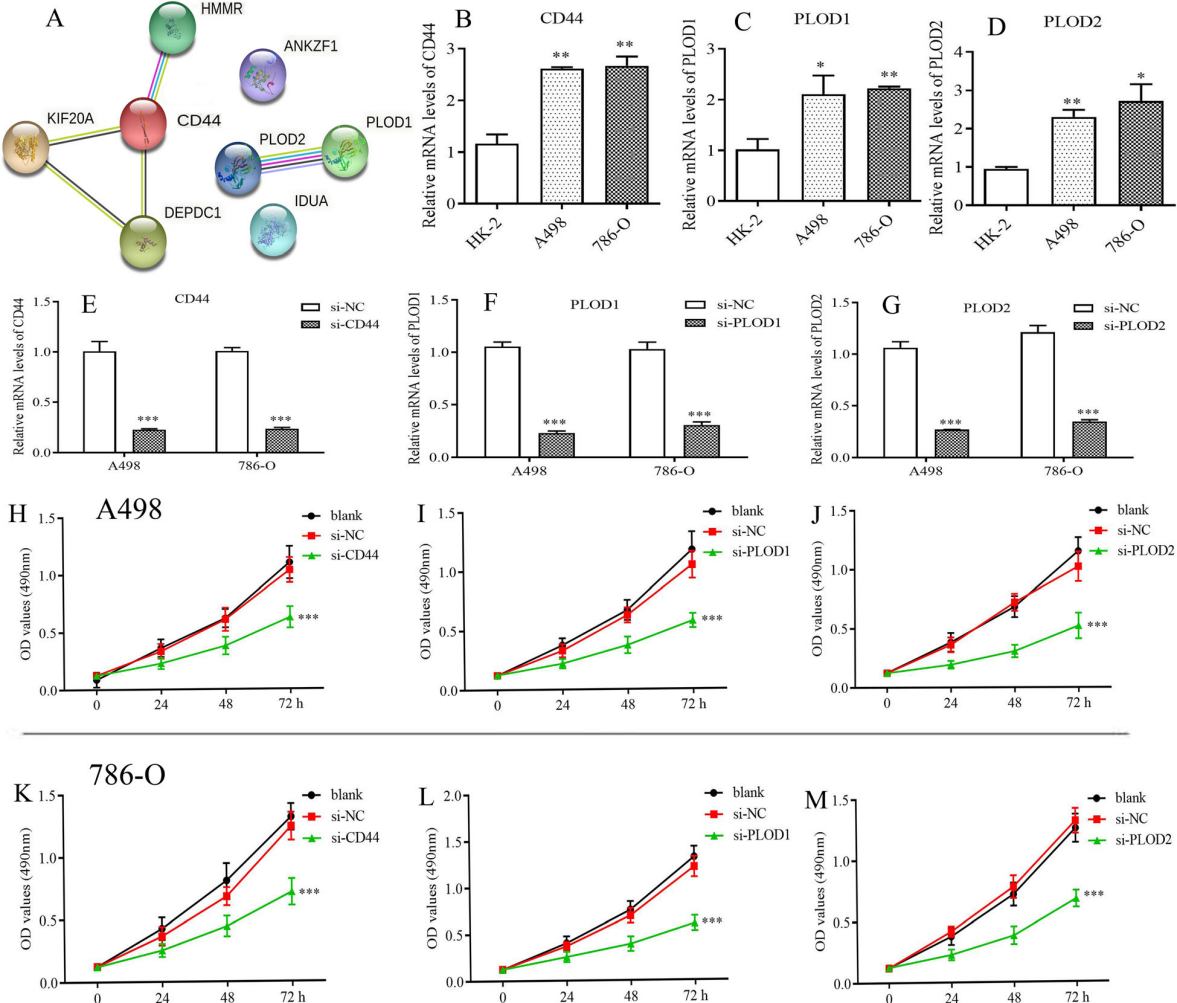
CD44, PLOD1 and PLOD2 can promote the proliferation of renal cancer cells. **a** The PPI network of eight glycolytic risk genes. **b**-**d** The mRNA differential expression of three glycolytic genes in different cell lines. **e**-**g** Specific siRNAs inhibit the expression of CD44, PLOD1 and PLOD2 in mRNA levels. **h-j** Silencing of CD44, PLOD1 and PLOD2 can suppress the proliferation of A498 cells. **k-m** Silencing of CD44, PLOD1 and PLOD2 can suppress the proliferation of 798-O cells. PPI, protein-protein interaction; NC, negative control; * means *p*<0.05; * * means *p*<0.01; * * * means *p*<0.001

The PPI network of eight glycolytic risk genes was constructed through the String database. As shown in Fig. 8a, CD44, PLOD1 and PLOD2 were in the central of the network suggesting these genes maybe the core genes among glycolysis-related risk signature. Therefore, we validated the biofunctions of CD44, PLOD1 and PLOD2 in renal cancer cells through further vitro experiments.

**Fig. 9:**
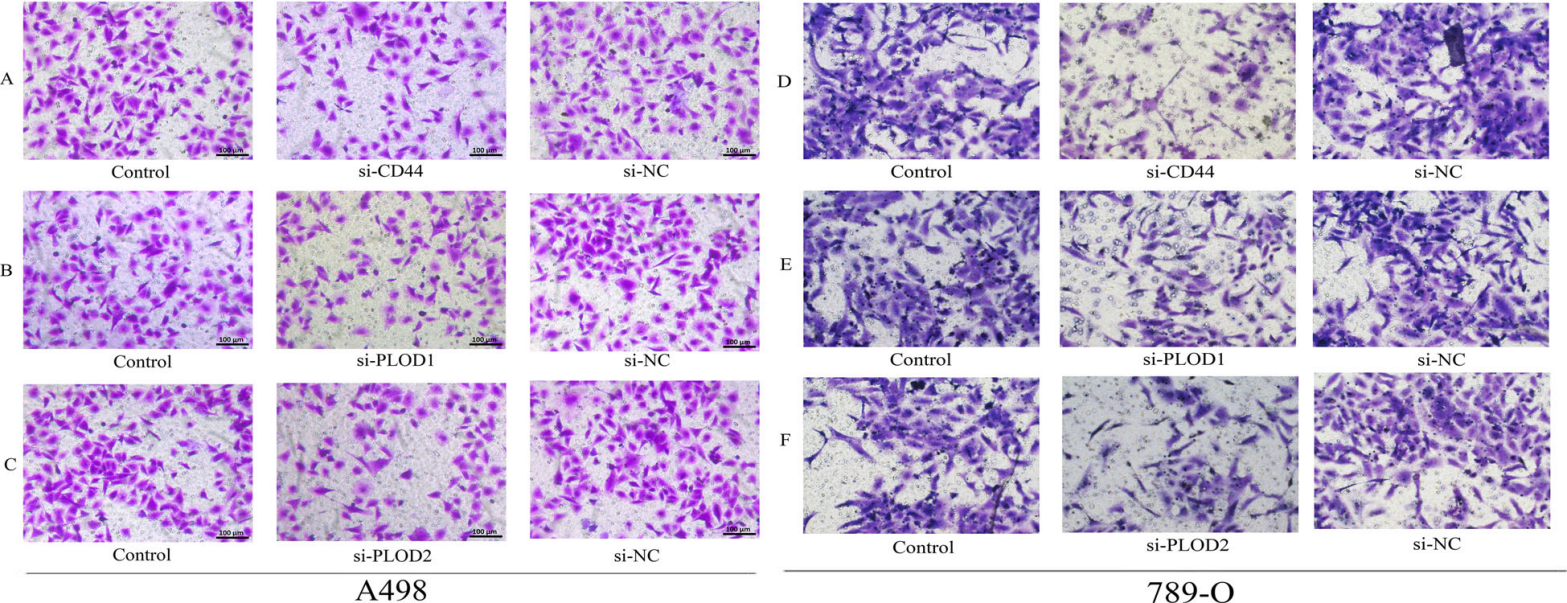
The assessments of invasive abilities of CD44, PLOD1 and PLOD2 through Transwell assays. **a**-**c** Knockdown of CD44, PLOD1 and PLOD2 can inhibit the invasion of A498 cells. **d**-**f** Knockdown of CD44, PLOD1 and PLOD2 can inhibit the invasion of 789-O cells. NC, negative control

Except for IDUA and HMMR, other six risk genes were all up-regulated in renal cancer cells (Fig. 8b-d and supplementary Figure 4). To investigate the biofunctions of CD44, PLOD1 and PLOD2 in RCC, we synthesized siRNAs of these genes to decrease their expression. The qPCR tests confirmed that their expressions were significantly down-regulated in transfected renal cancer cells (Fig. 8e-g). MTT and Transwell invasion assays were employed to evaluate the proliferative and invasive abilities of cells. As expected, empty vector transfection (NC group) did not affect cell viability. However, silencing of CD44, PLOD1 and PLOD2 inhibited the proliferative abilities of A498 and 789-O cells (Fig. 8h-m). Moreover, knockdown of CD44, PLOD1 and PLOD2 can suppress the invasion of A498 and 789-O cells, compared with those transfected with si-NC (Fig. 9). As a result, we ascertained that CD44, PLOD1 and PLOD2 played a pro-cancer role in RCC through vitro experiments, reiterating that glycolytic risk signature was closely associated with RCC progression.

## Discussion

Since Otto Warburg first discovered in 1924 that tumor cells preferred to engage in aerobic glycolysis, this cellular process has gradually been confirmed as one of the cancer hallmarks, that is also known as the Warburg effect [7]. In the present study, four glycolysis-related gene sets from the MSigDB were significantly enriched in RCC samples, suggesting that aerobic glycolysis is also prevalent in RCC. However, the reasons that tumor cells preferred to utilize glycolysis, an inefficient metabolic pattern, rather than oxidative phosphorylation for their proliferation, remain entirely unclear. Based on previous studies, we speculated there are three reasons for this tumoral metabolic preference.

First of all, proliferating cells have important metabolic requirements beyond ATP [38]. Cell proliferation requires a large number of nucleotides, amino acids, and lipids. A molecule of glucose provides a maximum of 36 molecules of ATP, but only 2 molecules of NADPH and 6 molecules of carbon [39]. During cell proliferation, massive glucose consumption is not a prerequisite for ATP production, but is essential for the synthesis of biological products that are needed for proliferation. For instance, during synthesizing the palmitate, an important component of cellular membranes, a molecule of glucose provides five times ATP as much as needed, but less than 20% carbon and NADPH as required [38]. Moreover, the pentose phosphate pathway, a branch from glycolysis, is the main source of nucleotides and NADPH during proliferation process [40]. Similarly, our KEGG analysis also indicated glycolytic genes significantly enriched in the pentose phosphate pathway. Meanwhile, NADPH supplied by the pentose phosphate pathway can protect cancer cells from oxidative stress [41]. Thus, the glycolytic metabolism is inefficient for ATP production but efficient for cell proliferation. Secondly, aerobic glycolysis can induce tumor cells’ therapeutic resistance. Classic chemotherapy treatments generally target rapidly dividing cells; however, quiescent tumor cell populations evade therapeutic destruction. It has been demonstrated that proliferating tumor cells can induce the accumulation of lactic acid through prompting glycolysis metabolism, which leads to cellular acidosis and quiescence [42, 43]. When external treatments are terminated, quiescent cells can reenter proliferative state and complete their therapeutic evasion [44]. Thirdly, glycolysis can maintain a high ratio of ATP/ADP, which leads to the continuous activation of cell proliferation [45, 46]. Although glycolysis is not a highly efficient ATP producer, it can regulate the activity of adenylate kinases to buffer the declining ATP production [38, 47]. In summary, the glycolytic metabolism can provide stimulating signals and a sufficient biomass for cell proliferation, and assist in tumor cells therapeutic resistance. The GSEA results also revealed that glycolysis is prevalent in RCC, which is consistent with previous studies [48].

After screening, we found that eight glycolytic risk genes were differentially expressed in RCC; however, their mutation frequency is not high (37/354, 10.5%). This suggests that the aberrant expression of these genes may be a result of post-transcriptional regulations or translation modifications. For example, over-expressed DEPDC1 is negatively regulated by miR-26b, which facilitates cell proliferation in triple negative breast cancer (TNBC) [49]. Moreover, the loss of the von Hippel-Lindau (VHL) gene is the most prominent genetic alteration in RCC, that is associated with over 80% of RCC cases [50]. Downregulated VHL elevated the accumulation of HIF-1 and led to the activation of hypoxia-inducible genes, such as PLOD1 and PLOD2, which is a key metabolic step for the transition into aerobic glycolysis [51–53]. Similarly, KEGG analysis also indicated that glycolytic genes are involved in the HIF-1 signaling pathway (Fig. 2d), suggesting that the screened genes may play a crucial role in glycolytic metabolic switch. Mutation analysis is valuable for the identification of tumorous susceptible genes, which can be used as favorable markers for early diagnosis and therapeutic target prediction [54]. In the present study, the highest mutational frequency among glycolytic risk genes was only 7%, which demonstrates that the glycolytic risk signature may not serve as RCC susceptibility genes.

With the development of high-throughput genomics, many genes have been identified as potential biomarkers of tumor prognosis and progression, and as therapeutic targets. In the present study, we constructed a glycolysis-related risk signature and validated the biofunctions of its hub genes (CD44, PLOD1 and PLOD2) in RCC. CD44, a non-kinase transmembrane glycoprotein, is regard as a marker of cancer stem cell (CSC) and can regulate the properties of CSCs, including cellular plasticity, self-renewal, invasiveness and treatment resistance [55]. Meanwhile, Gan Yu et al. demonstrated that miRNA-34a suppressed the proliferation and metastasis of renal cancer cells by inhibiting CD44 [56]. In the current research, silencing CD44 also can weaken proliferative and invasive abilities of renal cancer cells, which coincided with previous research [56]. PLOD1 and PLOD2 are the key enzymes of collagen synthesis and extracellular matrix formation, therefore can drive tumorigenesis and angiogenesis in many cancers [57]. Although PLOD1 was proven to promote aggressiveness of bladder cancer cells [58], the potential roles of PLOD1 and PLOD2 in RCC were not elucidated. Through MTT and Transwell invasion assays, we confirmed the pro-cancer abilities of PLOD1 and PLOD2, which provided new basis for the molecular mechanism of RCC tumorigenesis.

The glycolysis-related gene signature was confirmed as an independent prognostic factor of RCC and can distinguish the prognostic difference of the patients under the same clinical subgroup (Fig. 4). Therefore, we believed that the novel risk signature was valuable for RCC clinical assessment. Firstly, glycolytic risk signature is an important supplement to RCC prognosis analysis. Single TNM staging system cannot accurately predict RCC prognosis. Correa et al. have found that the predictive ability of TNM model was significant variable over time and its predictive accuracy was not satisfactory (C-index = 0.60) [59]. Nevertheless, as DCA curve displayed (Fig. 4b), taking glycolysis risk signature into the RCC prognostic analysis can add net benefit when making clinical decision. Secondly, dividing RCC patients into different risk groups drives individualized treatment strategies. When patients are classified into high-risk group, their follow-up time could be shortened and some adjuvant therapy could be attempted.

Tumor immune microenvironment commonly reflects the immune status of cancer, which could give support to formulate therapeutic strategy. CD8 T cells could differentiate into cytotoxic T cells, which exhibit cytotoxicity against tumor cells [60]. CD4 T cells could assist antitumor immunity and deficient CD4 T cells reduce the response of cytotoxic T lymphocytes (CTLs) [61]. Therefore, these two types of lymphocytes are the core ingredients for tumor cellular immunity. However, the glycolytic gene signature cannot affect infiltration levels of CD4 and CD8 T cells, which reveals that the impact of risk signature on immune microenvironment of RCC is limit. Besides, alterations of immune abundance caused an intricate effect on antitumor immune process (Table 4). For example, Mast cells are bidirectional to tumor progression [37]. On one hand, Mast cells promote cancer growth, stimulate neoangiogenesis and remodel tissue through releasing potent proangiogenic factors such as vascular endothelial growth factor (VEGF) and basic fibroblast growth factor (bFGF) [37]. On the other hand, they can suppress cell proliferation, inhibit immunologic stimulation and cell mobility by secreting chymase, tryptase, TNF-α, IL-1 and IL-6 [37]. Hence, we difficultly determine the final effect of decreasing mast cells (in high risk group) on antitumor immunity.

It’s believed that our findings could bring some advance to RCC field. Firstly, potential therapeutic targets. The role of Warburg’s effect in RCC has been widely confirmed and blocking the VHL-HIF-Glycolysis axis has been considered as a potential therapeutic strategy for renal cancer [62, 63]. For example, 2-deoxy-D-glucose, a glycolysis inhibitor, can not only kill various pathological subtypes of renal cancer cells, including clear cell RCC (ccRCC), papillary RCC and the rare subtype chromophobe RCC, but also enhance susceptibility of ccRCC to pazopanib treatment [64]. In the present study, we demonstrated that inhibitions of three core glycolytic genes suppressed the proliferation and invasion of renal cancer cells, indicating that these genes may serve as potential therapeutic targets of RCC. Secondly, assisting in prognostic analysis. Using TCGA database, we established a novel glycolysis-related prognostic model, which could assess the risk levels of RCC patients with the same TNM stage and contribute to make prognostic assessment more accurately. Thirdly, prompt for genetic regulatory mode. VHL mutations occur in more than 80% RCC cases [50]. Invalidation of VHL factor stimulates the expression of glycolytic genes and increases the glycolytic flux through stabilizing HIF [48]. However, in mutation analysis, we found that only 10.5% of RCC samples harbored with the mutations of glycolytic genes, suggesting that the ectopic expression of glycolytic genes in RCC may be accomplished by epigenetic modification rather than mutation. For example, miR-122-5p can promote proliferation and invasion of renal cancer cells by targeting PKM2 (a glycolytic limiting enzyme) [65]. LncARSR-miR-34a-5p-HK1 axis (HK is another glycolytic limiting enzyme) facilitates the progression of rectal cancer by promoting glycolysis metabolism [66]. In summary, our discoveries provide new insights into the roles of glycolytic genes in RCC from the perspectives of prognosis, molecular mechanism and regulatory mode.

Naturally, there are some limitations in our study. Firstly, the biofunctions of glycolysis-related genes in RCC have not been verified through vivo models and clinical samples. Secondly, glycolytic prognostic model should be tested in actual clinical cases. Thirdly, we did not assess the impact of fasting blood glucose (FBG) and anti-diabetes drugs on final results. According to some metabolomic discoveries and oncological researches, we found that some hypoglycemic drugs and patients’ FBG certainly regulated the tumor progression, which may affect the results of this study. For example, Metformin was proven to possess anti-cancer ability in acute myeloid leukemia (AML) [67] and lung cancer [68] by inhibiting glycolytic process. Ashton TM et al. suggested that Metformin may be a potential antitumor drug for its antagonistic action against oxidative phosphorylation (OXPHOS) and glycolysis [69]. Regarding FBG, Ayush Sharma et al. confirmed that high level of FBG was associated with large tumor volume and undesirable histological grade in pancreatic cancer [70]. In gastric cancer, elevated FBG and high SNHG8 expression resulted in poor survival outcomes after radical gastrectomy [71]. Unfortunately, TCGA database does not provide information on FBG and anti-diabetes drugs, which we believe is indeed one of the limitations of the present study.

## Conclusions

To the best of our knowledge, we constructed a novel glycolysis-related risk signature in RCC for the first time. The glycolytic risk signature played a crucial role in progression, prognosis and immune microenvironment of RCC. Moreover, three core glycolytic risk genes, CD44, PLOD1 and PLOD2, were capable of promoting the proliferation and invasion of renal cancer cells. Our findings can provide new inspirations for RCC prognostic analysis and treatment.

## Supplementary Information

**Additional file 1: Supplementary Figure 1.** Differential expression of eight risk signature genes in RCC samples. RCC, renal cell carcinoma.

**Additional file 2: Supplementary Figure 2.** The glycolysis-related risk signature could not distinguish the prognostic difference of RCC patients with G1, G4, N1 and T4 stages. RCC, renal cell carcinoma. G, grade.

**Additional file 3: Supplementary Figure 3.** The distribution of immune abundance of 22 leukocyte subtypes in each RCC samples. RCC, renal cell carcinoma.

**Additional file 4: Supplementary Figure 4.** The mRNA differential expression of five glycolytic genes in different cell lines. * means *p*<0.05; * * means *p*<0.01.

**Additional file 5: Supplementary Table 1.** The description of glycolysis-related gene sets.

**Additional file 6: Supplementary Table 2.** The detailed parameter settings of GSEA. GSEA, Gene-set enrichment analysis; MSigDB, The Molecular Signatures Database; NA, not applicable; Meandiv, Mean deviation.

**Additional file 7: Supplementary Table 3.** All primers for qRT-PCR.

## Data Availability

The datasets used and/or analyzed in the current study are available from the corresponding author upon reasonable request.

## Abbreviations

RCC: Renal cell carcinoma
TCGA: The Cancer Genome Atlas
GSEA: Gene-set enrichment analysis
OS: Overall survival
OSR: Overall survival rate
HK: Hexokinase
PFK: Phosphofructokinase
MSigDB: The molecular signatures database
NES: Normalized enrichment score
NOM: Nominal
FDR: False discovery rate
DEGs: Differentially expressed genes
PPI: Protein-protein interaction
Go: Gene ontology
KEGG: Kyoto encyclopedia of genes and genomes
MF: Molecular function
BP: Biological process
CC: Cellular components
HR: Hazard ratio
PLOD: Procollagen-lysine, 2-oxoglutarate 5-dioxygenases
HMMR: Hyaluronan mediated motility receptor
DEPDC1: DEP domain containing 1
IDUA: alpha-L-iduronidase
KIF20A: Kinesin family member 20A
VHL: Von Hippel-Lindau
CTLs: Cytotoxic T lymphocytes
HPA: The Human Protein Atlas
DCA: Decision curve analysis
NB: Net benefit
ROC: Receiver operating characteristic curve
