# Supplementary figures and images for: The effect of a novel glycolysis-related gene signature on progression, prognosis and immune microenvironment of renal cell carcinoma
Source: BMC Cancer. 2020 Dec 7;20:1207. doi: 10.1186/s12885-020-07702-7 (PMC7720455; doi:10.1186/s12885-020-07702-7)

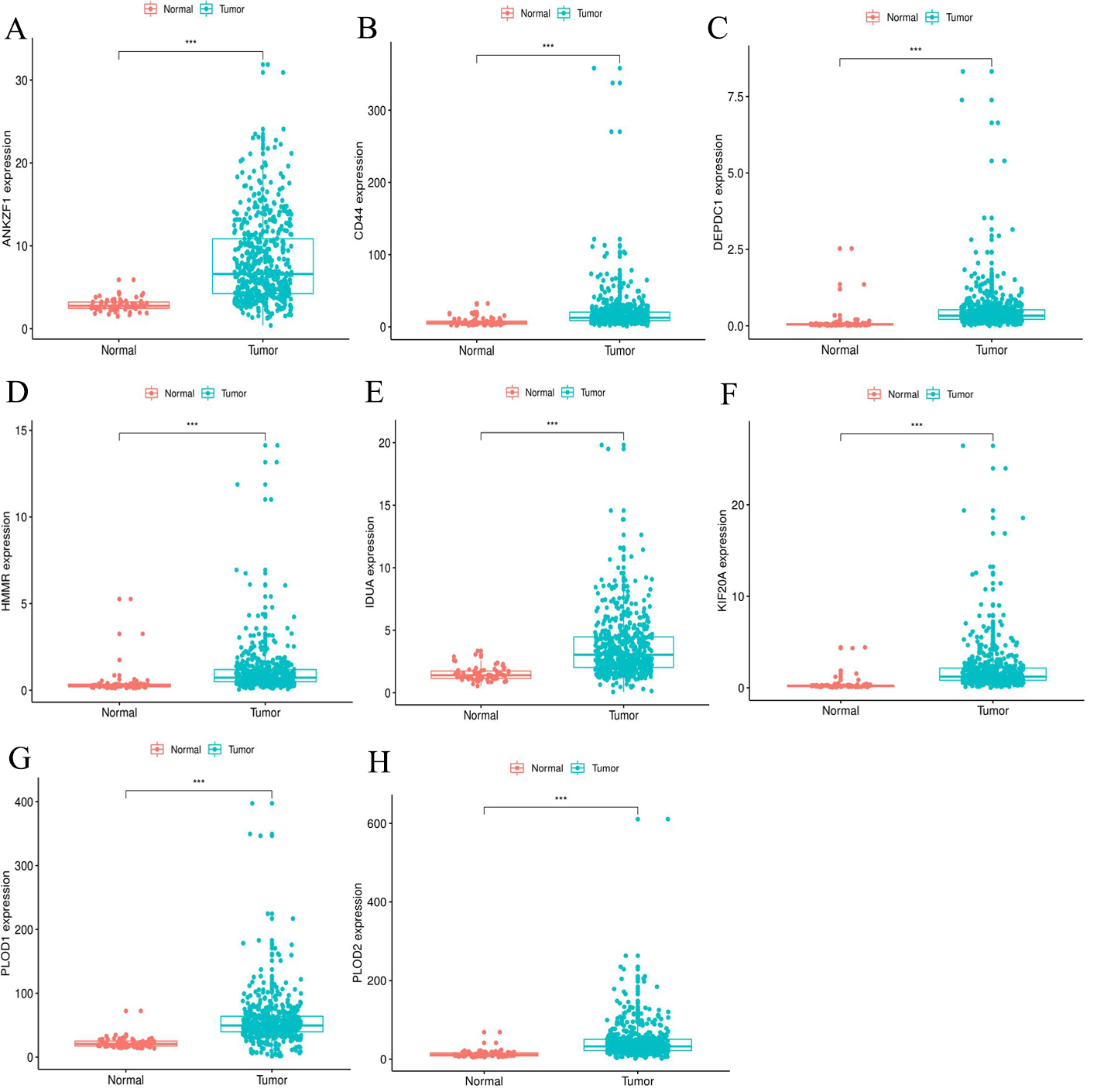

Supplement: Supplementary file 1 — Additional file 1: Supplementary Figure 1. Differential expression of eight risk signature genes in RCC samples. RCC, renal cell carcinoma. [file 12885_2020_7702_MOESM1_ESM.jpg]

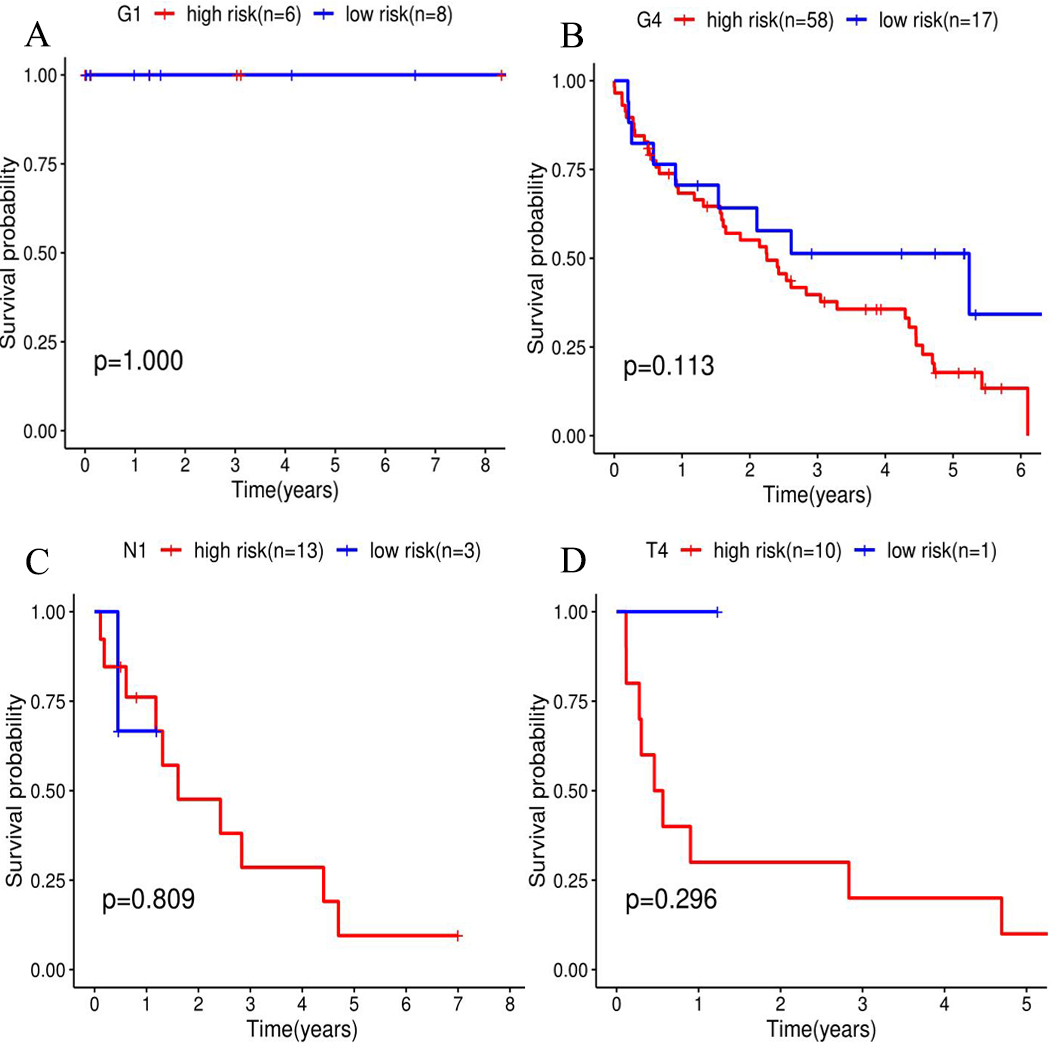

Supplement: Supplementary file 2 — Additional file 2: Supplementary Figure 2. The glycolysis-related risk signature could not distinguish the prognostic difference of RCC patients with G1, G4, N1 and T4 stages. RCC, renal cell carcinoma. G, grade. [file 12885_2020_7702_MOESM2_ESM.jpg]

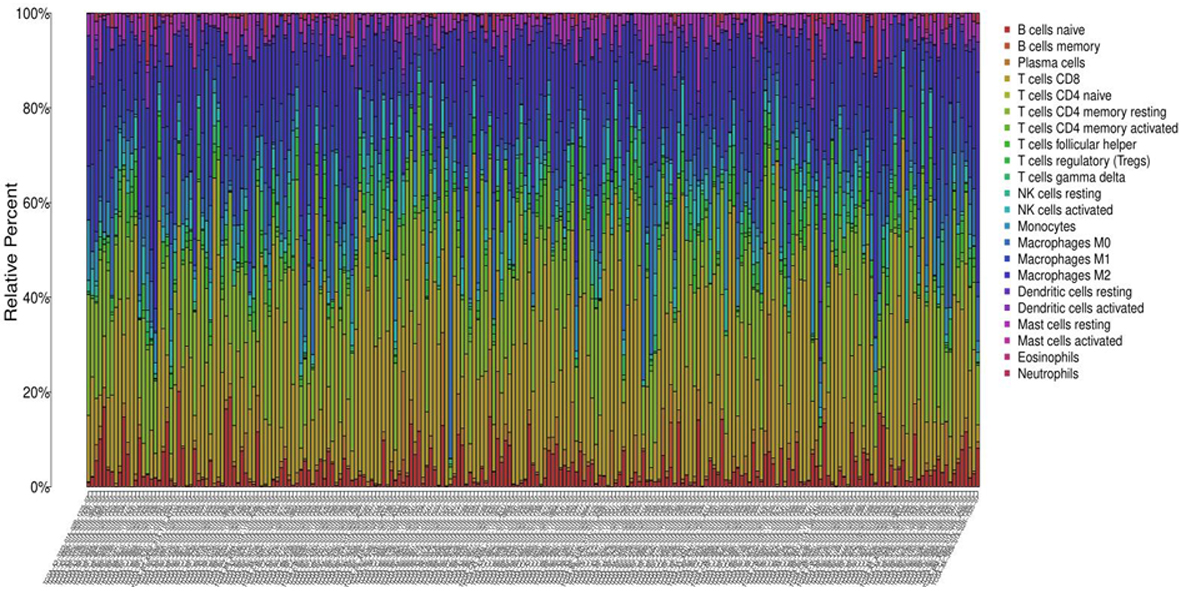

Supplement: Supplementary file 3 — Additional file 3: Supplementary Figure 3. The distribution of immune abundance of 22 leukocyte subtypes in each RCC samples. RCC, renal cell carcinoma. [file 12885_2020_7702_MOESM3_ESM.jpg]

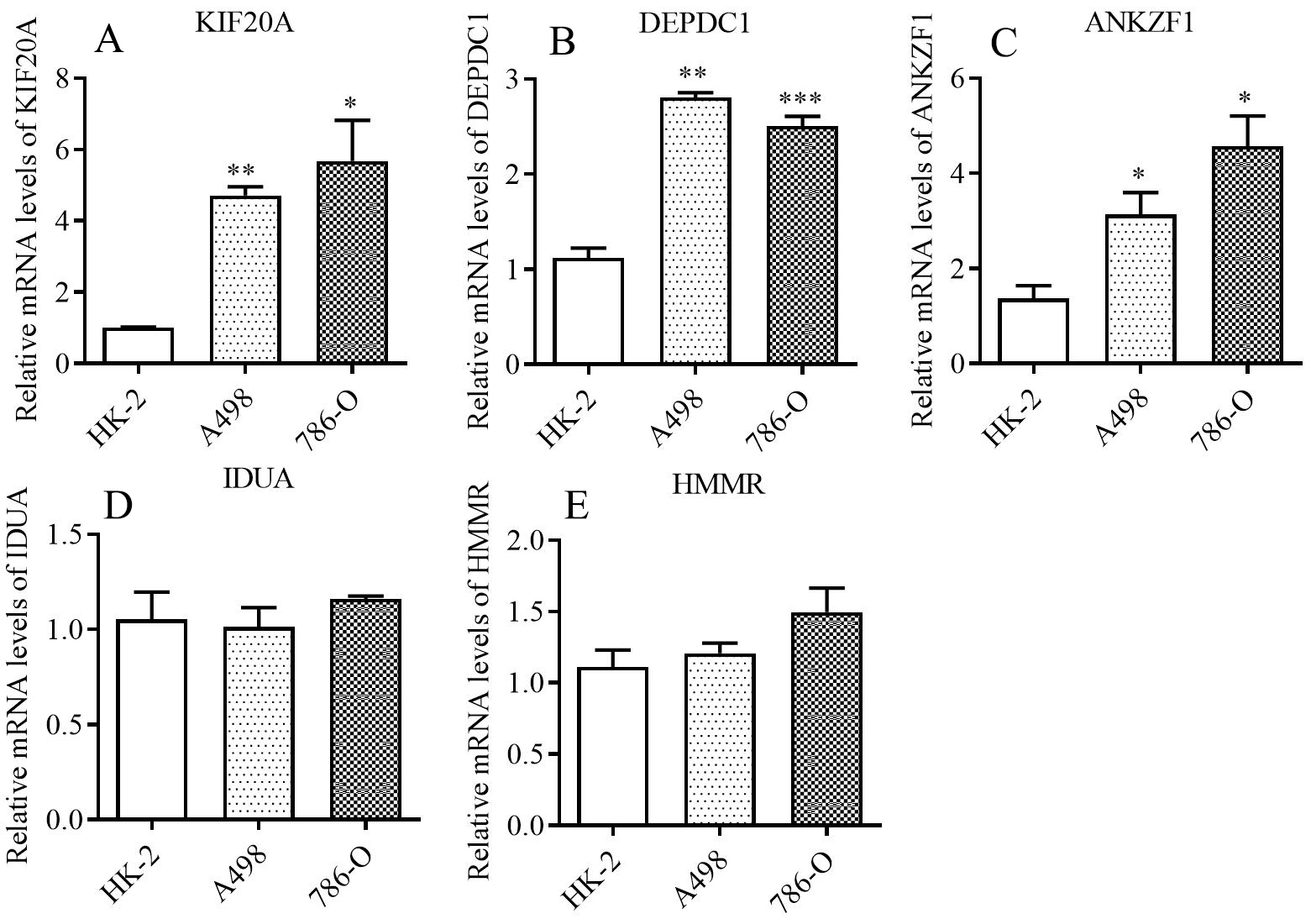

Supplement: Supplementary file 4 — Additional file 4: Supplementary Figure 4. The mRNA differential expression of five glycolytic genes in different cell lines. * means p<0.05; * * means p<0.01. [file 12885_2020_7702_MOESM4_ESM.jpg]
