# Supplementary material for: The effect of a novel glycolysis-related gene signature on progression, prognosis and immune microenvironment of renal cell carcinoma
Source: BMC Cancer. 2020 Dec 7;20:1207. doi: 10.1186/s12885-020-07702-7 (PMC7720455; doi:10.1186/s12885-020-07702-7)
Supplement: Supplementary file 5 — Additional file 5: Supplementary Table 1. The description of glycolysis-related gene sets. [file 12885_2020_7702_MOESM5_ESM.docx]

Supplementary Table III. All primers for qRT-PCR

| Gene | Primer | Sequence (5' -> 3') |
| --- | --- | --- |
| CD44 | Forward | CTGCCGCTTTGCAGGTGTA |
|  | Reverse | CATTGTGGGCAAGGTGCTATT |
| PLOD1 | Forward | AAGCCGGAGGACAACCTTTTA |
|  | Reverse | GCGAAGAGAATGACCAGATCC |
| PLOD2 | Forward | CATGGACACAGGATAATGGCTG |
|  | Reverse | AGGGGTTGGTTGCTCAATAAAAA |
| IDUA | Forward | CAGGAGATACATCGGTAGGTACG |
|  | Reverse | TCATGGAGACGTTGTCAAAGTC |
| HMMR | Forward | ATGATGGCTAAGCAAGAAGGC |
|  | Reverse | TTTCCCTTGAGACTCTTCGAGA |
| KIF20A | Forward | TCTGTCGTCTCTACCTCCCTA |
|  | Reverse | CAAGGGCCTAACCCTCAAGTA |
| DEPDC1 | Forward | TTTTGGTCCTGAAGTTACAAGGC |
|  | Reverse | TGGATACCTTCGTGGTAGAGTTT |
| ANKZF1 | Forward | GTTCAACTTGTGACCAGACCTT |
|  | Reverse | GCTTTAGGTTAAACCGATGCCA |
