# Supplementary material for: The effect of a novel glycolysis-related gene signature on progression, prognosis and immune microenvironment of renal cell carcinoma
Source: BMC Cancer. 2020 Dec 7;20:1207. doi: 10.1186/s12885-020-07702-7 (PMC7720455; doi:10.1186/s12885-020-07702-7)
Supplement: Supplementary file 6 — Additional file 6: Supplementary Table 2. The detailed parameter settings of GSEA. GSEA, Gene-set enrichment analysis; MSigDB, The Molecular Signatures Database; NA, not applicable; Meandiv, Mean deviation. [file 12885_2020_7702_MOESM6_ESM.docx]

Supplementary Table II. The detailed parameter settings of GSEA

| Parameter | Setting |
| --- | --- |
| Gene sets database | Seven glycolysis-related gene sets from MSigDB |
| Number of permutations | 1000 |
| Phenotype labels | Tumor samples versus normal samples |
| Collapse/Remap to gene symbols | No collapse |
| Permutation Type | Phenotype |
| Chip platform | NA |
| Enrichment statistic | Weighted |
| Metric for ranking genes | Signal2Noise |
| Gene list sorting mode | Real |
| Gene list ordering mode | Descending |
| Max size: exclude larger sets | 500 |
| Min size: exclude smaller sets | 1 |
| Collapsing mode for probe sets =＞1 gene | Max probe |
| Normalization mode | Meandiv |
| Randomization mode | No balance |
| Omit features with no symbol match | True |
| Median for class metrics | False |
| Number of markers | 100 |
| Plots graphs for the top sets of each phenotype | 20 |
| Seed for permutation | timestamp |
| Save random ranked lists | False |
| Make a zipped file with all reports | False |

GSEA, Gene-set enrichment analysis; MSigDB, The Molecular Signatures Database; NA, not applicable; Meandiv, Mean deviation.
