# Supplementary material for: The effect of a novel glycolysis-related gene signature on progression, prognosis and immune microenvironment of renal cell carcinoma
Source: BMC Cancer. 2020 Dec 7;20:1207. doi: 10.1186/s12885-020-07702-7 (PMC7720455; doi:10.1186/s12885-020-07702-7)
Supplement: Supplementary file 7 — Additional file 7: Supplementary Table 3. All primers for qRT-PCR. [file 12885_2020_7702_MOESM7_ESM.docx]

Supplementary Table I. The description of glycolysis-related gene sets

| Names | Gene counts | Description |
| --- | --- | --- |
| Hallmark Glycolysis | 200 | Genes encoding proteins involved in glycolysis and gluconeogenesis. |
| Go Glycolytic Process | 5 | Fermentation that includes the anaerobic conversion of glucose to pyruvate via the glycolytic pathway |
| KEGG Glycolysis Gluconeogenesis | 62 | Glycolysis / Gluconeogenesis |
| Reactome Glycolysis | 72 | Glycolysis |
| BioCarta Feeder Pathway | 9 | Feeder Pathways for Glycolysis |
| Biocarta glycolysis pathway | 3 | Glycolysis pathway |
| Module 306 | 26 | Glycolysis and TCA cycle. |
